# Complications and Outcome of Electrical Burns in Manipal, India: 6-Year Institutional Report

**DOI:** 10.29252/wjps.9.1.14

**Published:** 2020-01

**Authors:** Joseph Thomas, NC Sreekumar, Chandni Shankar, Alphy James

**Affiliations:** Department of Plastic Surgery, Kasturba Medical College Manipal, Manipal Academy of Higher Education, Manipal, Karnataka, India

**Keywords:** Electrical burn, Morbidity, Complication, India

## Abstract

**BACKGROUND:**

Electrical burns, although less prevalent, are devastating injuries and are associated with high morbidity and mortality. This study assessed the socio-demographic characteristics, complications, surgical interventions and outcomes among electrical burn victims.

**METHODS:**

From 2013 to 2018, patients who suffered from electric burns and were admitted to Burns Unit, Department of Plastic Surgery, Kasturba Hospital, Manipal, India were enrolled. The demographic data, as well as details regarding mode of injury, percentage of burns, specific areas injuries, complications, surgical treatment options utilized and treatment outcomes were recorded using a semi-structured questionnaire. The patients were followed up till 3 months post discharge.

**RESULTS:**

The majority of electrical burn victims were men (99.0%) and were in the age group of 18-40 years (70.4%). Unskilled labourers (56.8%) were most commonly affected followed by employed linemen or electricians (29.6%) and farmers (11.1%). Highest proportion (81.0%) had involvement of less than 20% of their total body surface area. Occurrence of infections (41.9%) was the most common complication. Myoglobinuria (19.7%), amputations (18.5%), compartment syndrome (14.8%), and peripheral nerve injuries (13.5%) were recorded. Totally, 18.5% were reported with certain complications, 9.9% of them required neurosurgical interventions and 3.7% required active psychiatric interventions.

**CONCLUSION:**

Most of the young men in their economically productive age group were affected with electrical burn injuries. Ensuring the work safety measures and education about the dangers and hazards associated with electrical equipment and infrastructure as well as their proper handling are vital.

## INTRODUCTION

Electrical burn injuries are a devastating form of burn injury.^1^^,^^2^ They constitute approximately 0.04–5% of admissions to burn units in developed countries, and up to 27% in developing countries.^3^ Even though they contribute only to a small share of all burn injuries, they are considered one of the most serious forms of injuries due to its high morbidity and mortality.^1^^,^^2^ In the adult population, electrical burn injuries primarily affect men, and are most often work-related, and are among the leading causes of traumatic work-related deaths. They tend to have physical as well as psychological short-term and long-term sequelae.^4^

Electrical injuries can be caused by flash, flame, lightning and true electrical injuries. Flash injuries, caused by an arc flash, are typically associated with superficial burns, as no electrical current travels past the skin. Flame injuries occur when an arc flash ignites an individual’s clothing, and electrical current may or may not pass the skin in these cases. Lightning injuries are associated with an electrical current flowing through the individual’s entire body due to extremely short but very high voltage electrical energy.^5 ^

True electrical injuries involve an individual becoming part of an electrical circuit. In these cases, an entrance and exit site is usually found.^5^ The severity of the injury depends on the intensity of the electrical current, the pathway it follows through the victim’s body and the duration of the contact with the source of the current.^6^ The burn injuries, as mentioned above varies with the intensity of the electrical current viz., high voltage (>1000 V) and low voltage (<1000V).^7^

Low voltage burn injuries are often limited to the area close to the sites of entry or exit. High voltage burn injuries are associated with an electrical current flowing through the individual’s entire body leading to necrosis of tissues often distant from the sites of entry and exit and maybe far more extensive than what it initially appears often requiring fasciotomies, extensive debridement and even amputations.^5^ Almost all electrical injuries are accidental and often, preventable. Presence of extensive burns and tissue necrosis, associated traumatic wounds, myocardial injuries and arrhythmias, neurological injury, secondary organ failures will determine the subsequent outcome and long-term prognosis.^6^

Early death usually occurs due to current-induced ventricular fibrillation or asystole, respiratory arrest secondary to paralysis of the central respiratory control system or due to paralysis of the respiratory muscles or major injuries secondary to trauma such a fall from height.^6^ Although advances in resuscitation and emphasis on early damage control surgeries, have improved clinical outcomes, fatalities are common. Thus prevention remains the best way to minimize the prevalence and severity of electrical injury.^6^ As the data on electrical injuries and its outcome remain scarce and varies with different inpatient settings, this study was conducted to assess the socio-demographic characteristics, complications and outcomes of electrical injuries among the study subjects.

## MATERIALS AND METHODS

In a longitudinal cross-sectional study for a period of nearly 6 years from 2013 to 2018, burns patients were enrolled who were admitted in the Burns Ward, Department of Plastic Surgery, Kasturba Hospital, Manipal, India as a tertiary care referral centre. The data was collected by reviewing the medical records of all the electrical burn patients admitted in the burns ward. The records with incomplete data were excluded from the study. After obtaining the ethical clearance from the institutional ethics committee, data was collected by the principal investigator using a semi-structured questionnaire which included the data on socio-demographic details, mode of injury, percentage of burns, specific sites of injury, complications, surgical treatment options utilized and their outcomes. 

All the victims were followed up at the end of one week, one month, three months post discharge and later when required. The modified Lund and Browders chart was used to assess the percentage of body surface area involved in the burn wound. Data was entered in Microsoft Excel sheet and the analysis was done using SPSS software (version 20.0, Chicago, IL, USA). Categorical data was expressed in terms of frequencies and percentages and continuous data was expressed in terms of mean±SD.

## RESULTS

A total of 81 cases of electrical burns were considered for the study among whom, men formed the majority (80, 98.8%). The economically productive age group between 18-40 years were the predominantly (57, 70.4%) affected ones. However, 3 (3.7%) of the cases were in the age group between 5-18 years. Among the victims, the majority were unskilled labourers (46, 56.8%) often employed temporarily or on contract basis dealing with power transmission infrastructures or factories using machineries requiring high voltage electricity.

Totally, 24 (29.6%) were formally employed by the government electricity board as linemen or electricians. The next group were farmers (9, 11.1%) who were accidentally injured while working in the fields having high tension electric wires running over them. Accidental entanglement of their tools onto low hanging wires was the common history noted. Hypertension, diabetes and epilepsy were the associated comorbidities in 11.1% (9/81) of the burn victims (Table 1).

**Table 1:** Socio-demographic characteristics of the study subjects

**Socio-demographic characteristics**	**Number of cases (n)**	**Percentage (%)**
Age group in years		
5 – 18 18-40 >40	03 57 21	3.7 70.4 25.9
Gender		
Males Females	80 01	98.8 1.2
Occupation		
Unskilled labourers Part-time/ Short informal contracts^§^ Employed full time^§^ Electricians/linemen Farmers Students Businessmen	46 40 6 24 9 1 1	56.8 87.0 13.0 29.6 11.11 1.2 1.2
Comorbidities		
Yes No	9 72	11.1 88.9

§- n=46

**Fig. 1:**
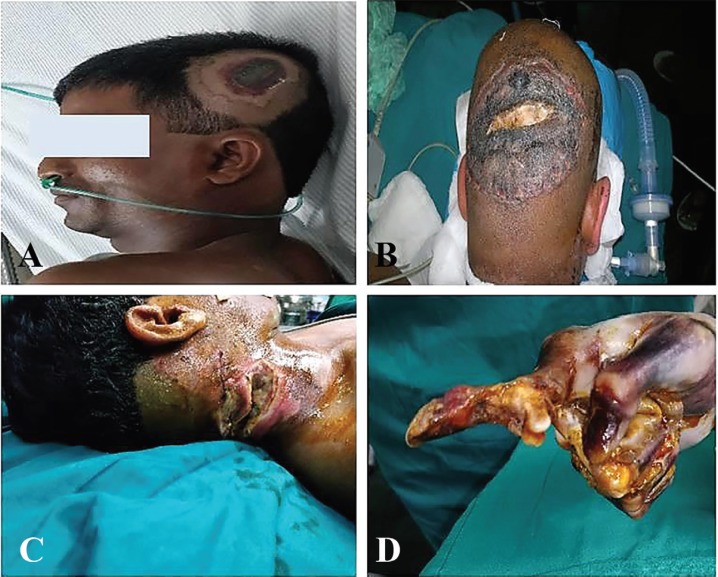
**A:** Scalp entry wound. **B:** Scalp entry wound with deep calcarial necrosis. **C:** Neck entry wound. **D:** Deccicated finger high voltage electrical injury

**Fig. 2:**
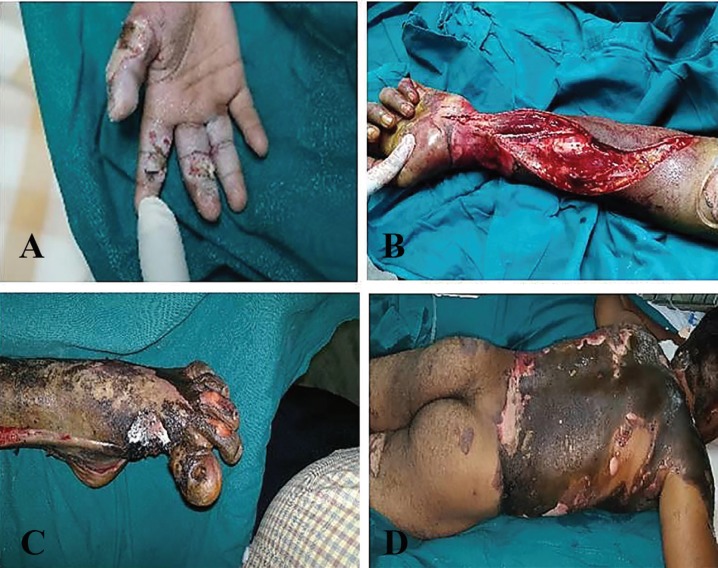
**A:** Low voltage electrical injury to finger. **B:** Decompression fascitomy for forearm. **C:** High voltage electrical injury **D:** Electrical flash burns injury to the back

**Fig. 3:**
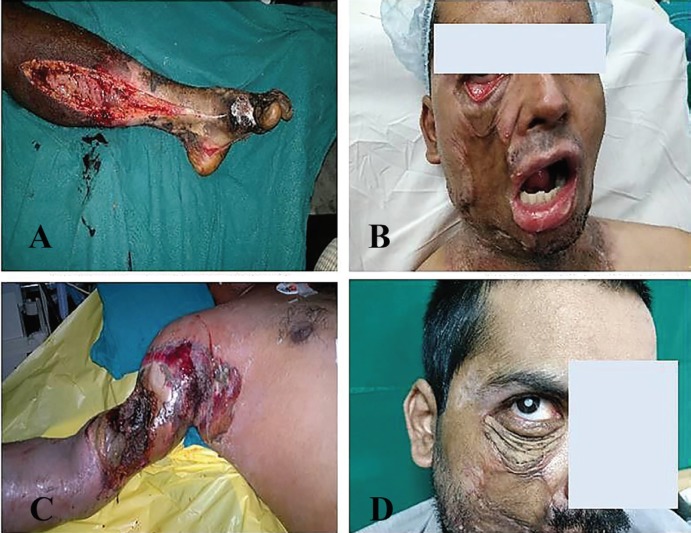
**A:** Decompression fasccotomy for leg. **B:** Lower eyelid ectropion. **C:** Electrical burn entry wound out the arm. **D: **Cataract following electrical burn injury

Nearly 35.0% (28/81) of the cases were associated with head injuries out of which 5 (17.9%) of them had electrical injuries to the scalp with calvarial involvement. Figure 1-3 demonstrate scalp, neck, eyelid, eye, finger, forearm, arm, back, and leg, electrical injuries. The highest proportion (66, 81.0%) of victims had involvement of less than 20% of their total body surface area and at least (3, 3.7%) had more than 50% of their total body surface area involved and 12 (14.8%) of victims had involvement of 20-50% of their total body surface area. 

Occurrence of infections (41.9%) was the most common complication followed by myoglobinuria (19.7%), amputations (18.5%), compartment syndrome (14.8%), and peripheral nerve injuries (13.5%) (Table 2). Nearly all cases (67, 82.7%) underwent debridement, including tangential excision (18, 22.2%). Split skin grafting was the most common (41, 50.6%) form of reconstruction, followed by suturing which was either done as a prima procedure or a delayed secondary one (38, 46.9%). Flap cover was provided in several cases (22, 27.2%). These included local transposition and rotation flaps for scalp injuries, fillet flaps and cross finger flaps for digits as well as regional abdominal flaps for upper limb cover. Explorative laparotomy was done for 2 (2.5%) patients one of which was for peritonitis secondary to ileal perforation.

**Table 2:** Complications of electrical burn injuries

**Complications**	**Number of cases (n) ** ^ᴥ^	**Percentage (%)** ^ᴥ^
Infections	34	41.9
Myoglobinuria	16	19.7
Amputations	15	18.5
Compartment Syndrome	12	14.8
Peripheral Nerve injuries	11	13.5
Renal failure	3	3.7
Long bone fractures	2	2.5
Intracranial bleeding	2	2.5
Osteomyelitis	2	2.5
Diffuse neurological deficits	2	2.5
Cardiac arrest	2	2.5
Intra-abdominal injuries	2	2.5
Ophthalmic injuries (Post electric burn cataract)	1	1.2
Vascular injuries (radial artery blowout)	1	1.2
Chest injuries (fractured rib with hemothorax)	1	1.2

^ᴥ^ n>81 and % >100 because of multiple responses

Additional surgeries performed included were penile reconstruction, ectropion correction, repair of bowel perforation, cataract surgery and abdominal wall repair. Out of the two cranioplasty procedures done, one involved creating drill holes in the calvarium (to encourage the growth of granulation tissue) and another involved full-thickness calvarial bone debridement and reconstruction with Poly-methyl methacrylate (PMMA) bone cement. Skin allografting was performed in 2 (2.5%) patients following wound debridement when extensive areas were involved due to flash burns (Table 3). 

**Table 3:** Surgical interventions among the study subjects

**Surgical interventions **	**Number of cases (n)** ^ᴥ^	**Percentage (%)** ^ᴥ^
Debridement	67	82.7
SSG	41	50.6
Primary/secondary suturing	38	46.9
Flap cover	22	27.2
Tangential excision	18	22.2
Fasciotomy	16	19.8
Escharotomy	3	3.7
Laparotomy	2	2.5
Allograft	2	2.5
Cranioplasty	2	2.5
Tissue expansion	1	1.2

^ᴥ^ n>81 and % >100 because of multiple responses

Mortality was reported among 8.6% (7) of the study subjects and those who survived were 74 (91.4%). Complications such as infected post-burn scar, osteomyelitis, persistent discharging sinuses, cataract, exposed calvaria, skin graft rejection, cellulitis, hypertrophic scar and keloid were reported in 15 (18.5%) cases. Also, 8 (9.9%) cases required neurosurgical intervention owing to various complications such as intracranial injuries, altered consciousness and sensorium, myelopathy of lower limbs, quadriplegia and subarachnoid haemorrhage and 3 (3.7%) of the patients needed active psychiatric interventions.

## DISCUSSION

Burn injuries can lead to a great burden in developing countries.^8^^,^^9^ Despite of significant progresses in burn management, burn injuries continue for a high toll, especially in predisposed patients and electrical burns.^10^ High energy current that travels through the body due to the contact with an electrical source lead to ‘electrical injuries’.^11^^,^^12^ It has been a significant cause of morbidity and mortality in both developing and developed countries around the world.^13^

Patients with electrical injury require immediate specialized care in order to minimize morbidity and mortality.^3^^,^^14^ Electrical injuries are known to affect the young population and work forces who form the main human resources of country. In a study by Ghavami *et al*., 97.8% of the affected victims were males and 59.4% were in the age group between 21-40 years similar to our study. The minor difference in the proportions of the age group affected compared to their study may be due to the difference in the inclusion of patients from 18 years in our study.^15^

Electrical injuries have a rare occurrence when compared to other types of burn injuries. Electrical injuries occurring among adults are considered an occupational hazard.^16^ In the same context, majority of our victims, were unskilled labourers (≈ 57.0%). Such unskilled personnel generally are inexperienced and handling electrical equipment by them, run a higher risk electrical burn injuries.^17^ But interestingly, 30.0% of the victims were formally employed by the government electricity board as linemen or electricians. Inadequate safety measures and training, lack of safety equipment and improper maintenance of infrastructure can lead to work place electrical injuries. 

According to Ghavami *et al*., about 60% of workers and electricians in their study declared that they were not aware of the dangers they may encounter while working with electrical devices.^15^ Farmers suffering electrical burns while working in the fields commonly gave a history of accidental entanglement of their tools onto low hanging wires. Eleven per cent of them were farmers who were accidentally injured while working in the fields having high tension electric wires running over them in our study. Agbenorku *et al*. found electrical burns casualties among self-employed was frequently due to non-adherence to safety precautions.^17^

Nearly 35.0% of the cases were associated with head injuries likely due to their falling down from high electric poles, and traumatic injuries from getting thrown by force due to the high voltage current in the wires. Nearly 18.0% of our subjects had entry/exit wounds on their scalp with calvarial involvement. Bhansali *et al*., reported 30-50% total body surface area involvement among maximum number of patients;^13^ but in our study, majority had involvement of less than 20% of total body surface area. 

In their study majority of the patients had suffered accidental thermal injuries due to various other reasons viz., stove blast, pressure cooker blast, clothes catching fire accidentally and kitchen gas leaks inclusive of electrical burns; however, our study was limited to electrical burn injuries wherein, only small entry and exit wounds would be seen masking the extensive internal organ injury or associated traumatic injuries that would have occured.^13^^,^^18^ Bounds *et al*. in their work noted that common complications following electrical injuries similar to those of other thermal burns, such as infection, compartment syndrome, and rhabdomyolysis which is comparable to our study findings with commonest complication being infections, myoglobinuria, amputations and compartment syndrome.^12^

Graft rejection, hypertrophic scar, keloid formation noted by Gajbhiye *et al*.,^15^ amputations, acute respiratory distress syndrome, acute renal failure requiring dialysis, infection and ventilator support noted by Srivastava *et al*.,^19^ peripheral nerve injuries, cataract, keratoconjunctivitis, acute renal failure, massive small bowel necrosis, stomach perforation, haemorrhage and persistent epilepsy noted by Haberal *et a1*.,^20^ were the complications reported in their study. Although some findings were different from our study due to different study settings, these are the common expected complications following electrical burn injuries.^20^

In the current study, surgical procedures involved in managing the electrical burns included debridement, fasciotomies, split skin grafting, primary or secondary suturing, flap cover and explorative laparotomy. Additional surgeries performed included penile reconstruction, ectropion correction, and repair of bowel perforation, cataract surgery, abdominal wall repair, cranioplasty and skin allografting. Few cases even required neurosurgical intervention and active psychiatric interventions at a later date. 

According to the findings of Gajbhiye *et al*., procedures undertaken for management of burn cases were comparable to this study like debridement in all the patients, surgical excision with split-skin grafting, fasciotomy and subsequently secondary suturing, amputation, and repair by flap.^16^ Whereas in a study conducted by Srivastava *et al*., the reconstructive procedures performed were early excision and skin grafting, and distant flaps such as groin and abdomen flaps, microvascular free flaps, ear reconstruction, rhinoplasty, scalp reconstruction and tendon reconstruction.^19^

Excluding few, most of the procedures noted in our study were similar. The difference may be due to varied severity of injuries, their anatomical location and presence of complications. Our study reported a mortality of 8.6% among the study subjects which is comparable to the study findings of Gajbhiye *et al*., who noted 8.16% mortality.^16^ The current record based study tries to describe the overall outcome of the electrical burn injury patients through follow up for short term sequelae and is one of the very few studies which have used modified Lund and Browder’s chart to classify the involvement of TBSA in electrical burns. However the pattern of the electrical burns, intensity and long term complications has not been elicited in the current study. 

The majority of electrical burn victims were men (99.0%) and were in the age group of 18-40 years (70.4%). Unskilled labourers (56.8%) were most commonly affected followed by formally employed linemen or electricians (29.6%) and farmers (11.1%). Highest proportion (81.0%) had involvement of less than 20% of total body surface area. Occurrence of infections (41.9%) was the most common complication followed by myoglobinuria (19.7%), amputations (18.5%), compartment syndrome (14.8%), and peripheral nerve injuries (13.5%).

Nearly all cases (82.7%) underwent some form of debridement. Split skin grafting was the most common (50.6%) form of reconstruction. Mortality was reported among 8.6% of the study subjects. During the follow up, 18.5% were reported with complications like cataract, exposed calvaria, infected post-burn scar, osteomyelitis, discharging sinuses, graft rejection, cellulitis, hypertrophic scar and keloid. Totally, 9.9% of the cases required neurosurgical intervention and 3.7% of the patients required active psychiatric interventions. 

From our study, we would like to recommend the policy makers to make a priority action on prevention of electrical burns with focus on the safety of workers. Improved safety measures such as repair and maintenance of improper electrical installations, such as low hanging wires, repair and maintenance of equipment and infrastructure must be taken up on a priority basis. Effective training and education for injury prevention by stressing on the safety precautions such as the use of personal protective equipment (insulated gloves and footwear) and following national electrical codes should be ensured among the handlers of electrical wires or appliances.

## CONFLICT OF INTEREST

The authors declare no conflict of interest.
